# Granulocyte and monocyte adsorptive apheresis ameliorates sepsis in rats

**DOI:** 10.1186/s40635-017-0129-2

**Published:** 2017-03-24

**Authors:** Shuai Ma, Qingqing Xu, Bo Deng, Yin Zheng, Hongyan Tian, Li Wang, Feng Ding

**Affiliations:** 1 Division of Nephrology & Unit of Critical Nephrology, Shanghai Ninth People’s Hospital, School of Medicine, Shanghai Jiaotong University, 639 Zhizaoju Road, Shanghai, 200011 China; 2 Division of Nephrology, Huashan Hospital, Fudan University, 12 Wulumuqi Zhong Road, Shanghai, 200040 China

**Keywords:** Sepsis, Inflammation, Granulocyte, Monocyte, Adsorption, Cellulose acetate

## Abstract

**Background:**

Overwhelming activation of granulocytes and monocytes is central to inflammatory responses during sepsis. Granulocyte and monocyte adsorptive apheresis (GMA) is an extracorporeal leukocyte apheresis device filled with cellulose acetate beads and selectively adsorbs granulocytes and monocytes from the peripheral blood.

**Methods:**

In this study, septic rats received the GMA treatment for 2 h at 18 h after cecal ligation and puncture.

**Results:**

GMA selectively adsorbed activated neutrophils and monocytes from the peripheral blood, reduced serum inflammatory cytokine expression, and seemed to improve organ injuries and animal survival. GMA potentially reduced lung injury by alleviating the infiltration of inflammatory cells and the secretion of cytokines.

**Conclusions:**

This study showed that selective granulocyte and monocyte adsorption with cellulose acetate beads might ameliorate cecal ligation and puncture (CLP)-induced sepsis and improve survival and organ function.

## Background

Sepsis is defined as life-threatening organ dysfunction caused by a dysregulated host response to infection [1]. Despite progress in antibiotics and critical care therapy, sepsis remains the most common cause of death in the intensive care unit [2]. Sepsis affects the immune system by directly altering the lifespan, production, and function of the effector cells responsible for homeostasis [3]. Additionally, innate and adaptive immune system cells play a critical role in the host response to sepsis [4]. Leukocytes, such as granulocytes and monocytes, are central to the inflammatory responses to infection, which involves phagocytosis, the production and release of reactive oxygen species and cytokines, and the adhesion to and infiltration into the tissue [3–6]. However, overwhelming activation of granulocytes and monocytes can contribute to significant cell and tissue damage [7].

Different therapeutic approaches to prevent such damage caused by granulocytes and monocytes have been developed, such as inhibiting granulocyte and macrophage extravasation and adhesion, blocking the actions of their proinflammatory cytokines, and attenuating reactive oxygen species [8, 9]. However, these treatments have shown little or modest effects on disease process when tested clinically [8, 9]. Because activated leukocytes are central to the pathogenesis and progression of sepsis, an alternative approach to limit the deleterious clinical effect of activated leukocytes may be selective adsorption of excess and activated leukocytes, which may result in immunomodulation.

Granulocyte and monocyte adsorptive apheresis (GMA) is an extracorporeal leukocyte apheresis device filled with cellulose acetate beads and selectively adsorbs granulocytes and monocytes from the peripheral blood [10]. Cellulose acetate beads have been used to treat patients with various autoimmune diseases such as rheumatoid arthritis [11, 12], ulcerative colitis [10, 13], and Crohn’s disease [14]. The mechanism of selective adsorption is thought to be mediated by the interaction of the complement receptor and Fc receptor on granulocytes and monocytes, whereas complement factors and immunoglobulins adhere to the surface of cellulose acetate beads via ligand-receptor interactions [6, 15]. Typically, the beads adsorb approximately 65% of granulocytes, 55% of monocytes, and 2% of lymphocytes from the blood [16]. However, GMA has not yet been clinically applied to treat sepsis. Only one ex vivo animal study showed that selective adsorption regulated phagocytic activity and adhesiveness of granulocytes in sepsis [17] and thus, its effects remain unclear. In this study, we established an in vivo miniature GMA for rats to evaluate the therapeutic effects in sepsis.

## Methods

### Cecal ligation and puncture (CLP)

Adult male Sprague–Dawley rats (450–550 g) were used in this study. All surgical procedures were carried out under general anesthesia induced by 4% chloral hydrate (0.9 mL/100 g intraperitoneally). The cecum was identified and ligated at 25% length of the cecum. A double puncture of the cecal wall was performed with a 20-gauge needle, and the cecum was gently squeezed to ensure that a small amount of feces was extruded onto the surface of the bowel [18]. For sham CLP rats, the cecum was minimally handled without ligation and puncture.

### Experimental protocol for GMA use in rats

**Fig. 1:**
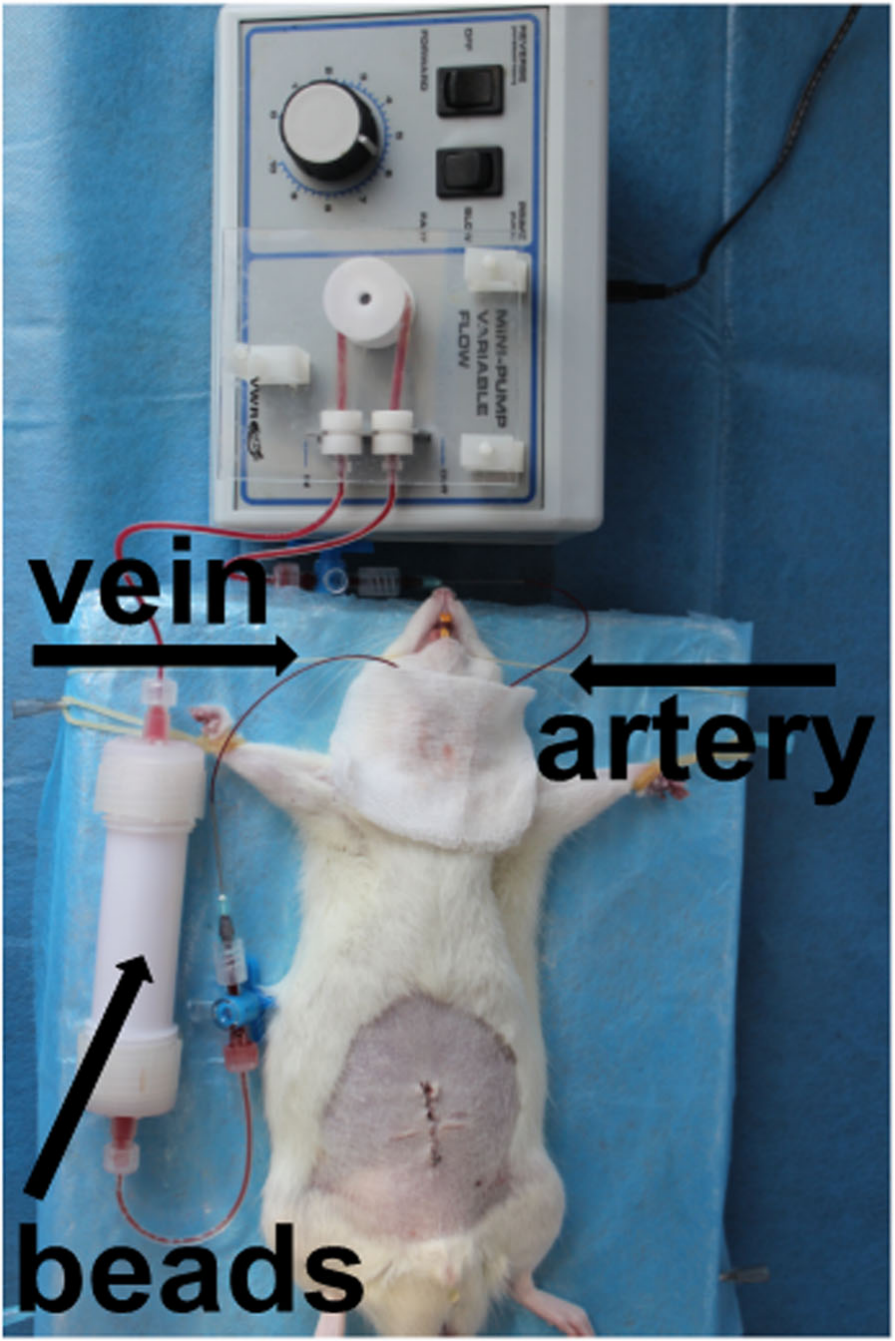
Schematic drawing of granulocyte and monocyte adsorptive apheresis (GMA) for septic rats

Eighteen hours after CLP, the animals were re-anesthetized with chloral hydrate. The left carotid artery and right external jugular vein were isolated by dissection and cannulated with 0.97-mm polyethylene-50 tubing (BD Biosciences, Franklin Lakes, NJ, USA) for implementation of extracorporeal circulation. The GMA consisted of a mini-column filled with 10 g cellulose acetate beads (cellulose acetate beads; JIMRO, Gunma, Japan), mini-pumps (VWR, West Chester, PA, USA), and tubing lines (Fig. 1).

Eighteen hours after CLP, the animals were randomly assigned to receive either GMA or sham GMA treatment for 2 h. Extracorporeal circulation was driven by a mini-pump from the left carotid artery to the right external jugular vein at a blood flow rate of 1.0 mL/min. A volume of 62.5 U/mL of heparin was used to prevent coagulation in this circuit. In the GMA group, the extracorporeal circulation passed through a mini-column filled with cellulose acetate beads. In the sham GMA group, the extracorporeal circulation was set up in the same manner but without beads. In the control group, all rats underwent the sham CLP procedure without extracorporeal circulation.

Blood samples were collected for cell classification and counting at 0, 1, and 2 h during extracorporeal circulation. Neutrophil and monocyte numbers were determined from stained cytospins by the Wright-Giemsa staining (Sigma-Aldrich, St. Louis, MO, USA). Survival time was assessed for up to 7 days.

### Scanning electron microscopy (SEM) and immunofluorescence (IF) images

After GMA treatment, the cellulose acetate beads were collected for observing the cell adherence to them using SEM and characterizing the adherent cells using IF microscopy. The beads were fixed in 1% OsO_4_, dehydrated in alcohol gradients, and then imaged using QUANTA 200 SEM (FEI, Hillsboro, OR, USA) as described previously [19]. For IF images, the beads were stained with DAPI (4′,6-diamidino-2-phenylindole; BD Biosciences) and CD11b (R&D Systems, Minneapolis, MN, USA) as described previously [19] and then observed under a confocal laser scanning microscope (Leica Microsystems, Heidelberg, Germany).

### Flow cytometric analysis

Blood samples were collected, and erythrocytes were lysed (Erythrocyte Lysis Kit; R&D Systems). Splenocytes and pulmonary cells were isolated as previously described [20, 21]. Briefly, spleens were harvested and homogenized by smashing with the plunger from a syringe. The dispersed spleens were passed through a 70-mm nylon mesh, and erythrocytes were lysed. The lungs were harvested, finely minced, and digested in 10 mL collagenase type II (2 mg/mL; Sigma-Aldrich), and then, erythrocytes were lysed.

Fluorochrome-labeled antibodies CD4, CD8, CD45, CD11b, HIS36, and HIS48 (eBioscience, San Diego, CA, USA) were used for surface staining according to the manufacturer’s instructions. After staining, the cells were analyzed by flow cytometry (BD Biosciences).

### Analysis of bronchoalveolar lavage fluid (BALF)

BALF was obtained by lavaging the left lung once with 2 mL PBS and three times with 4 mL PBS. Total cells were counted under optical microscopes. Total protein in the first BALF was measured using a bicinchoninic acid kit (Sigma-Aldrich). The levels of inflammatory-associated cytokines in the first BALF were measured by enzyme-linked immunosorbent assay (ELISA).

### ELISA

The concentrations of TNF-α, IL-1β, IL-6, and IL-10 were measured using ELISA kits from eBioscience (eBioscience, San Diego, CA, USA).

### Histopathologic analysis of the lung and liver

Lung sections were stained with hematoxylin and eosin. All lung fields at ×200 magnification were examined for each sample. Assessment of histological lung injury was performed as follows: 1 = normal; 2 = focal (<50% lung section) interstitial congestion and inflammatory cell infiltration; 3 = diffuse (>50% lung section) interstitial congestion and inflammatory cell infiltration; 4 = focal (<50% lung section) consolidation and inflammatory cell infiltration; 5 = diffuse (>50% lung section) consolidation and inflammatory cell infiltration [22].

Liver sections were stained with hematoxylin and eosin. Hepatic injury scores were measured based on liver morphological criteria [23]: spotty necrosis, capsular inflammation, portal inflammation, ballooning degeneration, and steatosis. The scoring system was as follows: 0 = none (no change), 1 = mild changes, 2 = moderate changes, 3 = severe changes.

### Measurements of liver functions

Serum alanine aminotransferase (ALT) and aspartate aminotransferase (AST) were measured with an automated chemical analyzer (Vitros-950, Johnson & Johnson, New Brunswick, NJ, USA).

### Statistical analysis

All numerical data are expressed as mean ± SEM. Student’s *t* tests were used for comparisons between two groups. Multiple group comparisons were performed by one-way ANOVA followed by a post hoc Tukey’s test to compare each group. Survival analysis was performed using the Kaplan-Meier method and log-rank test. All statistical analyses were performed with GraphPad Prism software (GraphPad Software, La Jolla, CA, USA), and a 2-sided *p* < 0.05 was considered significant.

## Results

### GMA selectively adsorbed peripheral blood leukocytes and ameliorated CLP-induced sepsis

**Fig. 2:**
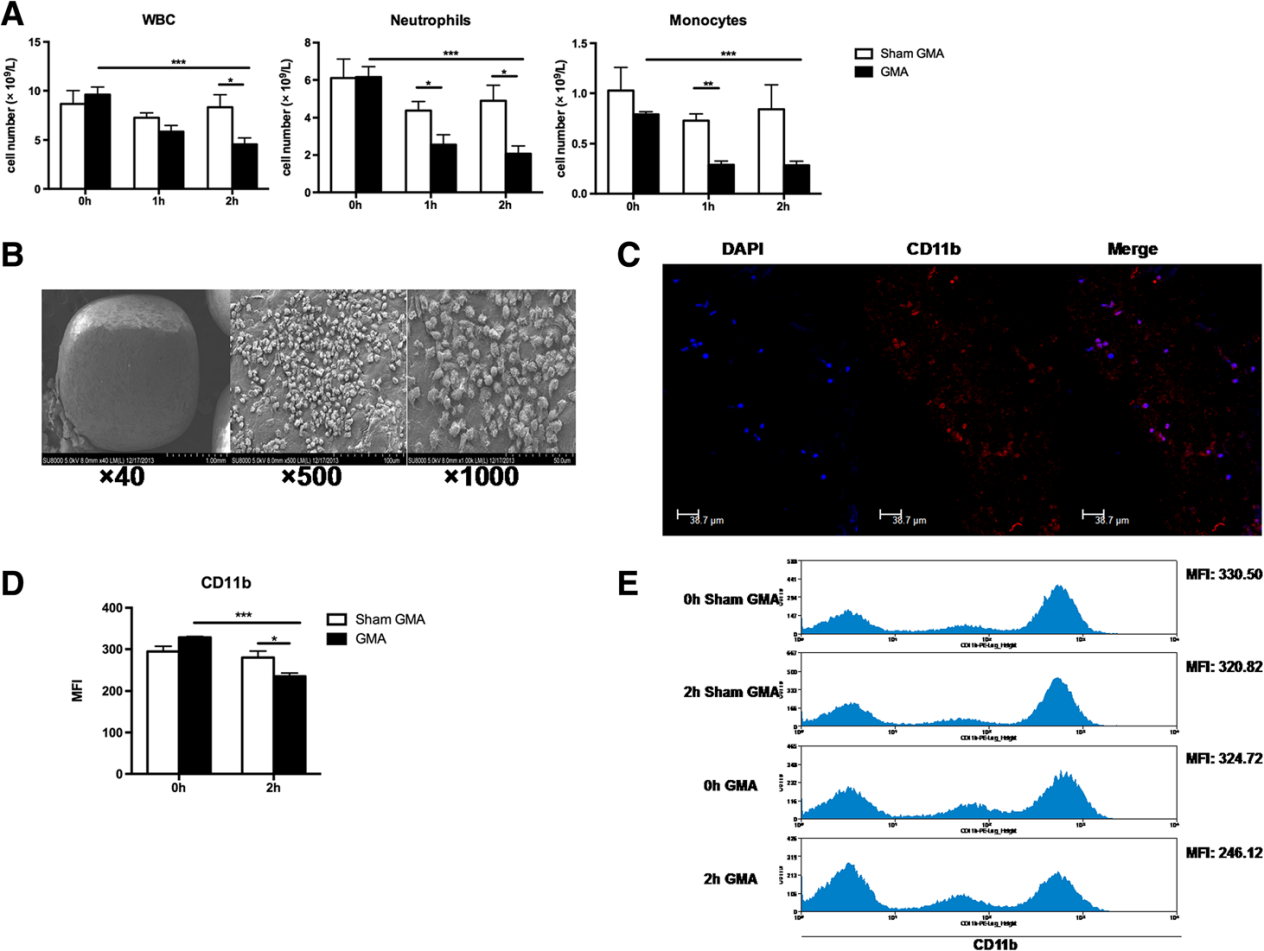
Granulocyte and monocyte adsorptive apheresis (GMA) selectively adsorbed activated neutrophils and monocytes of peripheral blood in septic rats. **a** White blood cells were counted at 0, 1, and 2 h during the GMA treatment; neutrophil and monocyte numbers were determined from stained cytospins by the Wright-Giemsa staining (*n* = 5–6 per group). **b** Scanning electron microscopy images of the cellulose acetate beads after GMA treatment. **c** Leukocytes were adsorbed onto the cellulose acetate beads after GMA treatment and were stained with DAPI (*blue*) and CD11b (*red*) at ×200 ×Z 1.0 magnification. **d**, **e** Peripheral blood neutrophils were collected at 0 and 2 h during GMA treatment. The mean fluorescence intensity of CD11b was analyzed by flow cytometry (*n* = 5–6 per group). *p < 0.05, **p < 0.01, ***p < 0.001. Data are expressed as mean ± SEM. *WBC* white blood cell, *DAPI* 4′,6-diamidino-2-phenylindole

After 2-h GMA treatment, the number of peripheral leukocytes significantly decreased (*p* < 0.001, 0-h GMA vs. 2-h GMA, Fig. 2a), while there was no change in the sham GMA group (*p* > 0.05, 0-h sham GMA vs. 2-h sham GMA, Fig. 2a). Particularly, this treatment resulted in the removal of neutrophils and monocytes from the peripheral blood (both *p* < 0.001, 0-h GMA vs. 2-h GMA, Fig. 2a). SEM and IF images confirmed the adsorption neutrophils and monocytes onto the surface of the beads (Fig. 2b, c). Mac-1 (CD11b/CD18) is expressed predominantly on neutrophils and monocytes/macrophages; when these cells are activated, Mac-1 expression increases [24]. Figure 2d, e shows the change in CD11b expression in peripheral leukocytes before and after GMA treatment. The expression of CD11b was significantly downregulated after GMA treatment compared with that after sham GMA treatment (*p* < 0.001, GMA vs. sham GMA, Fig. 2d, e). These data demonstrate that GMA might selectively adsorb activated neutrophils and monocytes in the peripheral blood.

**Fig. 3:**
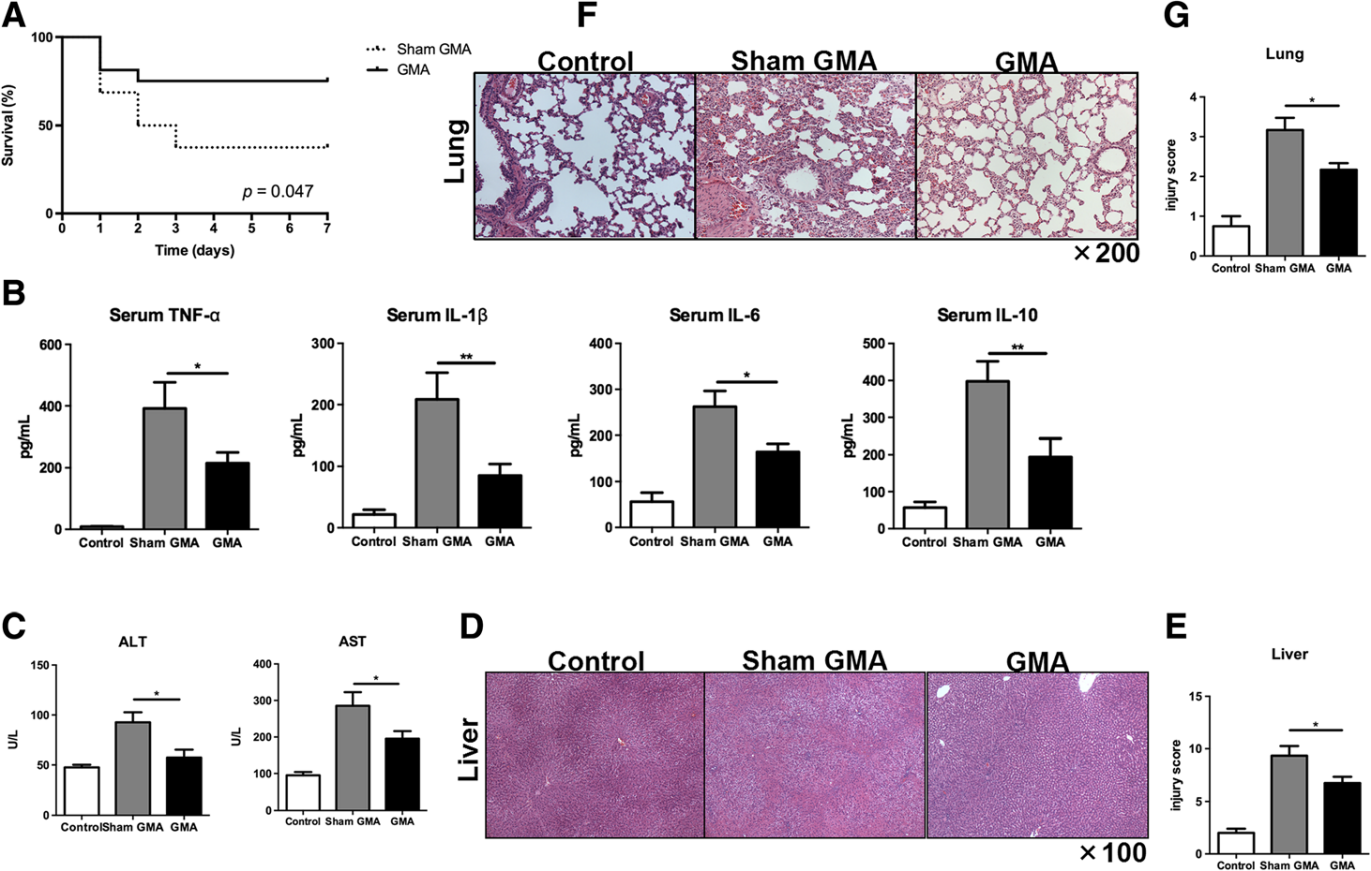
Granulocyte and monocyte adsorptive apheresis (GMA) ameliorated CLP-induced sepsis. **a** Survival was plotted for a 7-day period (*n* = 22–25 per group). **b** Serum inflammatory-associated cytokines were measured by ELISA at 48 h after CLP (*n* = 6–7 per group). **c** Serum ALT and AST were quantified at 48 h after CLP (*n* = 6–7 per group). **d**, **f** Representative photomicrographs of liver sections stained with hematoxylin and eosin (HE) and examined at ×100 magnification and of kidney sections stained with HE and examined at ×200 magnification. **e**, **g** Histopathologic mean liver and lung injury were scored at 48 h after CLP (*n* = 6 per group; at least 10 fields were reviewed for each slide). *p < 0.05, **p < 0.01. Data are expressed as mean ± SEM. *CLP* cecal ligation and puncture, *ALT* alanine aminotransferase, *AST* aspartate aminotransferase, *ELISA* enzyme-linked immunosorbent assay

We next investigated whether GMA treatment can ameliorate sepsis. First, we found that GMA improved the survival of rats with CLP-induced sepsis. The data showed that the 7-day survival rate of the GMA group was 46.7%, while the rate reduced to 73.3% in the sham GMA group (*p* = 0.047, GMA vs. sham GMA, Fig. 3a). Leukocytes release cytokines during sepsis. We next measured the levels of inflammatory-associated cytokines including TNF-α, IL-1β, IL-6, and IL-10 in the serum. At 48 h after CLP, the levels of these cytokines increased significantly in the sham GMA group compared with that in the control group (*p* < 0.001, Fig. 3b). GMA treatment decreased the levels of TNF-α (*p* < 0.05), IL-1β (*p* < 0.01), IL-6 (*p* < 0.05), and IL-10 (*p* < 0.01) compared with the subjects in the sham GMA treatment (Fig. 3b). Sepsis may result in multiple organ dysfunctions; thus, we assessed liver and lung injury. At 48 h after CLP, the levels of serum ALT and AST were elevated in the sham GMA group compared with the subjects in the control group (*p* < 0.05, sham GMA vs. control, Fig. 3c). These values decreased significantly in GMA-treated rats (*p* < 0.05, GMA vs. sham GMA, Fig. 3c). The pathology of the liver, which includes necrosis, inflammation, degeneration, and steatosis, in the sham GMA group showed much more severe injuries compared with that in the control group (Fig. 3d). However, the injuries were ameliorated after GMA treatment (Fig. 3d). Furthermore, injury scores in the liver (*p* < 0.05, sham GMA vs. GMA, Fig. 3e) confirmed the protective effects of GMA. The pathology of interstitial congestion and inflammatory cell infiltration in the lung indicated much more severe injuries in the sham GMA group than those in the control group (Fig. 3f). The injuries were ameliorated after GMA treatment (Fig. 3f). Similarly, after GMA treatment, the injury scores of the lung also decreased (*p* < 0.05, GMA vs. sham GMA; Fig. 3g). These data suggest that GMA treatment might prolong survival time, reduce inflammation, and ameliorate liver and lung injuries of CLP-induced sepsis compared with the sham GMA treatment.

### GMA alleviated pulmonary inflammation

**Fig. 4:**
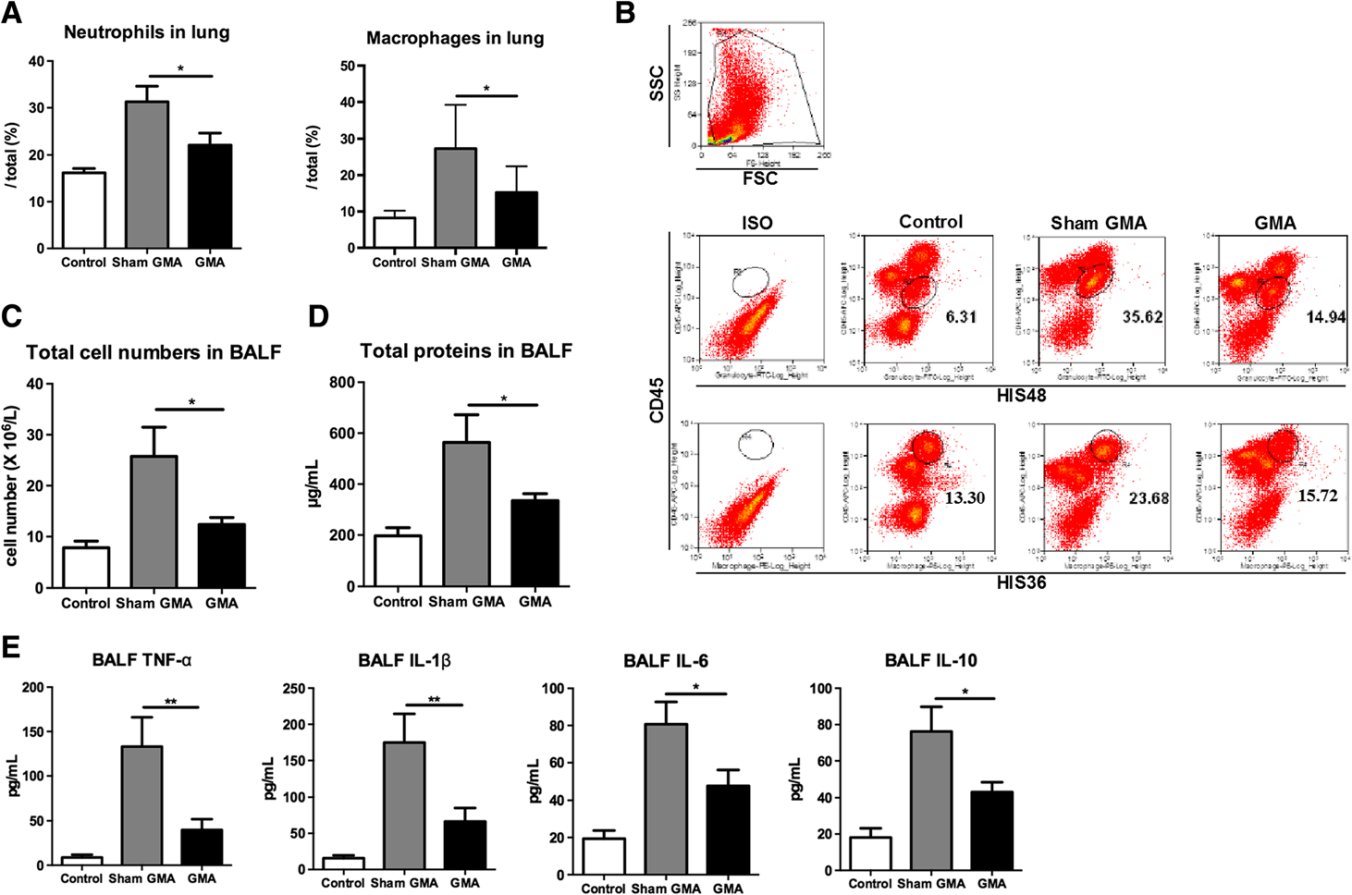
Granulocyte and monocyte adsorptive apheresis (GMA) alleviated inflammation in the lungs. **a** The levels of neutrophils and macrophages in the lung were analyzed by flow cytometry at 48 h after CLP (*n* = 7–9 per group). **b** Gating strategy for identifying neutrophils and macrophages in the lung. Granulocyte marker [HIS48] and CD45 double-positive cells were defined as neutrophils. Macrophage marker [HIS36] and CD45 double-positive cells were defined as macrophages of rats. **c** Total cell counts in BALF at 48 h after CLP (*n* = 7–9 per group). **d** Total protein in BALF at 48 h after CLP (*n* = 7–9 per group). **e** Inflammatory-associated cytokines in the BALF were measured by ELISA at 48 h after CLP (*n* = 7–9 per group). *p < 0.05, **p < 0.01. Data are expressed as mean ± SEM. *CLP* cecal ligation and puncture, *BALF* broncho alveolar lavage fluid, *ELISA* enzyme-linked immunosorbent assay

Inflammatory cells and cytokines lead to lung injury in sepsis; the lung is one of the first organs to be affected in this pathophysiological process [25]. Neutrophils and monocytes in the peripheral blood were adsorbed during GMA treatment. Thus, we analyzed whether this treatment could further reduce the infiltration of leukocytes in the lung using flow cytometry. The infiltration of neutrophils and macrophages in the lung of the GMA group was significantly reduced compared with that in the sham GMA group (both *p* < 0.05, Fig. 4a, b) at 48 h after CLP. We also counted the total cells in the BALF of different groups and found that the total BALF cell count in the sham GMA group was much higher than that in the GMA group (*p* < 0.05, Fig. 4c). We determined the concentration of total protein in the BALF to assess pulmonary vascular leakage. At 48 h after CLP, total protein levels in the BALF were elevated in the sham GMA group compared with the subject in the control group (*p* < 0.05, sham GMA vs. control, Fig. 4d), while total protein decreased significantly after GMA treatment (*p* < 0.05, GMA vs. sham GMA, Fig. 4d). To further evaluate pulmonary inflammation in septic rats, the levels of inflammatory-associated cytokines (TNF-α, IL-1β, IL-6, and IL-10) were measured in the BALF samples. We found that the levels of these cytokines were increased significantly in the sham GMA group compared with those in the control group (all *p* < 0.001, Fig. 4e). GMA treatment decreased the levels of these cytokines compared with the sham GMA treatment at 48 h after CLP (all *p* < 0.05, Fig. 4e). Taken together, these data suggest that the GMA might reduce lung injury by alleviating inflammatory cells and cytokines in the lung compared with the sham GMA treatment.

### No change in the level of T lymphocytes after GMA

**Fig. 5:**
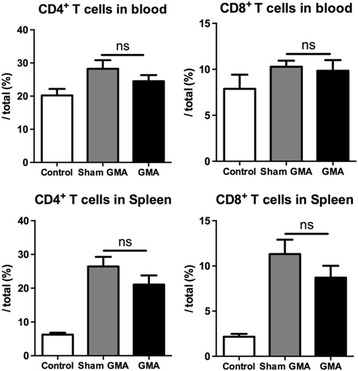
Granulocyte and monocyte adsorptive apheresis (GMA) had no effect on the levels of T lymphocytes in septic rats. Circulating and splenic levels of CD4^+^ helper T cells and CD8^+^ cytotoxic T cells were analyzed by flow cytometry at 72 h after CLP (*n* = 7–9 per group). *CLP* cecal ligation and puncture, *ns* no significance

To assess the adaptive immune response, circulating and splenic levels of CD4^+^ helper T cells and CD8^+^ cytotoxic T cells were measured by flow cytometry at 72 h after CLP. However, there was no significant difference in the peripheral blood or spleen between the GMA group and sham GMA group (all *p* > 0.05, Fig. 5). Thus, 2-h GMA treatment had no effect on the levels of T lymphocytes in the current rat model.

## Discussion

In the present study, we established a granulocyte and monocyte adsorptive apheresis for septic rats. The number of granulocytes and monocytes were decreased after passing through the cellulose acetate beads. Most adsorbed leukocytes were granulocytes. According to studies on ulcerative colitis, the reduction in granulocyte count peaked at 15 min after GMA treatment but did not decrease to below the normal range [17]. We also found that there was no significant decrease in the number of neutrophils after 4-h/6-h GMA treatment (data not shown). It is thought that neutrophils are mobilized from the marginal pools, including the bone marrow [26]. Some studies have shown reduced peripheral neutrophils during the GMA procedure, mainly old and activated neutrophils which were CD10^+^ [27]. The maximum mobilization of CD10^−^ neutrophils was observed within 30 min of GMA. CD10^−^ neutrophils are immature naïve neutrophils from the bone marrow and thought to not be proinflammatory [26].

It is thought that cellulose acetate beads can adsorb not only excess leukocytes but also activated leukocytes. Mac-1 (CD11b/CD18), the major subtype of integrins, is responsible for the firm adhesion of neutrophils to the endothelium. Once neutrophils are activated, the shape of these cells changed and the amount of Mac-1 increases, resulting in enhanced adhesion to the endothelium and transmigration and infiltration of these neutrophils [28]. In our experimental system, expression of CD11b on peripheral leukocytes was significantly downregulated after GMA treatment. Hara et al. also reported that the number of CD11b^+^ cells markedly decreased in an ex vivo study on septic models [17]. However, in inflammatory bowel disease and rheumatoid arthritis patients who received GMA treatment [24, 29], CD11b was upregulated, while L-selectin was downregulated. Upregulation of Mac-1 is not sufficient to promote leukocyte trafficking in these diseases because the reduction in L-selectin and the initial step in leukocyte-endothelial cell interaction is impaired; thus, the overall adhesiveness of granulocytes was reduced [24, 29].

Sepsis may result in multiple organ dysfunctions, such as injury of the lung, kidney, and liver. The septic response may be accelerated following continued activation of neutrophils and macrophages/monocytes [30], which migrate to damaged sites and induce cytokine release. In our study, after the adsorption of activated leukocytes, the levels of serum inflammatory-associated cytokines (TNF-α, IL-1β, IL-6, and IL-10) decreased and organ injuries were ameliorated. In some experimental and clinical studies have reported that the reduction or clearance of inflammatory mediators by various forms of blood purification such as high-volume hemofiltration, high-cutoff hemofiltration, and hemoadsorption in sepsis could improve organ functions [19, 31–34]. In another group, Zhiyong Peng et al. found that the CytoSorb beads could modulate leukocyte trafficking in a septic rat model and in an ex vivo human sepsis study [19, 35]. Those beads significantly removed plasma cytokines and chemokines and decreased BALF-to-blood chemokine ratios, resulting in decreased leukocytes recruitment into the lung. So, the modulation of chemokine gradients is important for leukocyte trafficking to different compartments during sepsis.

During bacterial infections, extensive interactions occur between antigen-presenting cells and lymphocytes, which are key effector cells in the adaptive immune response [30]. Sepsis not only causes hyper-inflammation but also leads to an anti-inflammatory or immunosuppressive phase. In this phase, the adaptive immune system is dysfunction, including immune cell depletion (especially T cells), compromised T cell functions and T cell exhaustion [36, 37]. To assess the adaptive immune response, we measured circulating and splenic levels of CD4^+^ helper T cells and CD8^+^ cytotoxic T cells at 72 h after CLP. However, the data showed that the 2-h GMA treatment had no effect on T lymphocyte levels.

This study had some limitations. First, our results for leukocyte surface marker (CD11b) expression reflected only the remaining circulating cells and not those adsorbed onto the beads. Moreover, L-selectin is involved in the initial step of the leukocyte-endothelial cell interaction. The levels of L-selectin need to be measured, which is important for evaluating the adhesiveness of granulocytes. Second, we were unable to determine whether this cell adsorption affected the adaptive immune response for not evaluating the function of lymphocytes. Third, further studies on different therapeutic doses and different onset times after CLP are needed.

## Conclusions

In summary, this is the first study to show that selective granulocyte and monocyte adsorption with cellulose acetate beads might ameliorate CLP-induced sepsis and improve survival and organ function in vivo compared with the sham GMA treatment. Additional studies are needed to further investigate these results. Our results may be used in the development of new therapies for patients with sepsis.

## Abbreviations

ALT: Alanine aminotransferase
AST: Aspartate aminotransferase
BALF: Bronchoalveolar lavage fluid
CLP: Cecal ligation and puncture
DAPI: 4′,6-Diamidino-2-phenylindole
ELISA: Enzyme-linked immunosorbent assay
GMA: Granulocyte and monocyte adsorptive apheresis
HE: Hematoxylin and eosin
IF: Immunofluorescence
SEM: Scanning electron microscopy
WBC: White blood cell
